# The Spectral Irradiance, Growth, Photosynthetic Characteristics, Antioxidant System, and Nutritional Status of Green Onion (*Allium fistulosum* L.) Grown Under Different Photo-Selective Nets

**DOI:** 10.3389/fpls.2021.650471

**Published:** 2021-03-25

**Authors:** Song Gao, Xuena Liu, Ying Liu, Bili Cao, Zijing Chen, Kun Xu

**Affiliations:** ^1^College of Horticulture Science and Engineering, Shandong Agricultural University, Tai’an, China; ^2^Collaborative Innovation Center of Fruit & Vegetable Quality and Efficient Production in Shandong, Tai’an, China; ^3^Key Laboratory of Biology and Genetic Improvement of Horticultural Crops in Huanghuai Region, Ministry of Agriculture and Rural Affairs, Tai’an, China; ^4^State Key Laboratory of Crop Biology, Tai’an, China

**Keywords:** green onion (*Allium fistulosum* L.), photo-selective nets, photosynthesis, product quality, plant antioxidant system

## Abstract

The active regulation of the plant growth environment is a common method for optimizing plant yield and quality. In horticulture today, light quality control is carried out using photo-selective nets or membranes to improve the yield and quality of cultivated plants. In the present study, with natural light as the control (CK), we tested different photo-selective nets (white, WN; blue, BN; green, GN; yellow, YN; and red, RN) with 30% shade for characteristics of growth, development, quality, yield, photosynthesis, and chlorophyll fluorescence, considering the antioxidant system, as well as the influence of element absorption and transformation of green onion (*Allium fistulosum* L.) plants at different growth stages. We found that plants under BN and WN have greater height and fresh weight than those of plants under the other nets. Plants under the BN treatment had the highest quality, yield, photosynthetic pigment content, net photosynthetic rate, transpiration rate, and stomatal conductance, whereas the intercellular CO_2_ concentration was the highest in plants in the YN treatment. The photosynthesis noon break phenomenon was significantly lower in plants with covered photo-selective nets than in CK plants. NPQ was the highest in the YN treatment, and Fv/Fm, ΦPSII, and qP among the plants in the other treatments were different; from highest to lowest, they were as follows: BN > WN > CK > RN > GN > YN. The active oxygen content of green onion leaves in the BN treatment was significantly lower than that in the other treatments, and their key enzyme activity was significantly increased. BN also improved the absorption and transformation of elements in various organs of green onion.

## Introduction

Light is an indispensable environmental factor in the life cycle of plants and is the main factor driving plant photosynthesis (Das, 2004). Irradiation quality, quantity, and photoperiod can regulate plant growth and development, trigger physiological and morphological reactions, and affect their secondary metabolism (Kurepin et al., 2007; Jackson, 2009; Wang et al., 2018a; Yangen et al., 2019). Under appropriate light conditions, plants can increase their CO_2_ fixation capacity and net photosynthetic rate by increasing the absorption of light energy and electron transfer rate (Yang et al., 2018). However, excessive light inhibits photosynthesis and can even lead to the photooxidation of photosynthetic organs (Wang et al., 2018b). The response of different species to light varies greatly (Lusk and Pozo, 2002).

The effects of global warming have already begun to have a significant impact on our environment, including an increase in atmospheric CO_2_ concentration, which may change the internal quality of vegetable products or lead to a decrease in photosynthesis rate (Bisbis et al., 2018). Among others, the challenges brought upon by global warming include an increase in air temperature (AT) and solar radiation intensity (Hu et al., 2018). For this reason, active manipulation of the plant growth environment is often used to optimize plant yield and quality (Dueck et al., 2016). With the adjustment of industrial structure and the continuous development of facility agriculture, photo-selective nets or films have been increasingly used in crop greenhouse technology to control the quality and quantity of ambient light to improve the yield, quality, and phytochemical composition of cultivated plants and protect them against pests and physical damage (Nissim-Levi et al., 2014; Selahle et al., 2014; Carvalho et al., 2016; Abbasnia Zare et al., 2019).

Photo-selective nets are a covering net material developed by the Israeli Agricultural Research Organization, Volcani Center, and Polysack Plastics Industries in Israel in the 1990s (Shahak et al., 2004). Compared with ordinary nets, certain pigments or light scattering and reflection aids are added to photo-selective nets in the production process, which is why the light quality changes after passing through the net. These nets can change the spectral composition of light, increase the proportion of scattered light (Shahak, 2008), and change the quality and quantity of light, as well as affect the airflow, temperature, and humidity.

Numerous studies have shown that different colors of photo-selective nets have different effects on plant growth, as well as that different plants have distinct responses to photo-selective nets. Compared to the use of black nets, the use of red nets led to an increase in the production of sweet peppers (Shahak, 2008; Darázsi, 2014). Furthermore, the pearl color net increased the number of branches of the pot plant *Myrtus communis* (Ada et al., 2015). However, research on *Pittosporum tobira* showed that the number of branches on stems increased most obviously under red nets, followed by gray nets, whereas it increased the least under black and blue nets (Stamps and Chandler, 2008). Experiments on basil plants showed that after covering the plants with red and blue nets (shading level 50%) for 90–120 days, leaf thickness and stomata density decreased compared to those in plants grown in the open field (Costa et al., 2010). Compared with red and black nets, blue nets increased the biomass of *Phalaenopsis* leaves and roots (Leite et al., 2008).

Green onion (*Allium fistulosum* L.) is often consumed as a vegetable or spice condiment and has high nutritional value (Gao et al., 2021). As the most important seasoned vegetable in Asia during summer, its seedlings are easily affected by high light. If the light is too strong, the leaf fiber increases, and the leaf body ages, which reduces its edible value and quality. Therefore, it is necessary to assess the growth of green onion during midsummer. In our experiment, through studying photosynthesis, chlorophyll fluorescence characteristics, resistance, quality, and yield of green onion leaves under photo-selective net conditions, we gained an in-depth understanding of the photosynthesis reaction mechanism of green onion under different spectral conditions and shades, which provides a theoretical basis for the cultivation of green onion in summer.

## Materials and Methods

### Plant Material and Sample Preparation

The experiment was conducted in the experimental field of Shandong Agricultural University (SDAU, N361.8°, E117.1°), Tai’an City, China, in 2019 based on a preliminary test in 2018. Green onion (*Allium fistulosum* L.) variety “Yuanzang” was originally obtained from the Tai’an Taishan Seed Industry Technology Co., Ltd. The seeds were sown on March 20, and the seedlings were about 40 cm high on June 25. They were moved to a planting ditch when they had four unfolded true leaves. The test soil was loamy with a pH of 6.72 and organic matter content of 11.09 g/kg.

In cultivation, row spacing was 80 cm, and plant spacing was 3.5–4 cm. A tent covering method was adopted for plant cultivation, that is, photo-selective nets (Fuzhou Kangsheng Knitting Textile Co., Ltd.) were placed directly on the support structure upper part of the green onion. The support structures were 2 m high, 10 m wide, and 20 m long.

**Figure 1:**
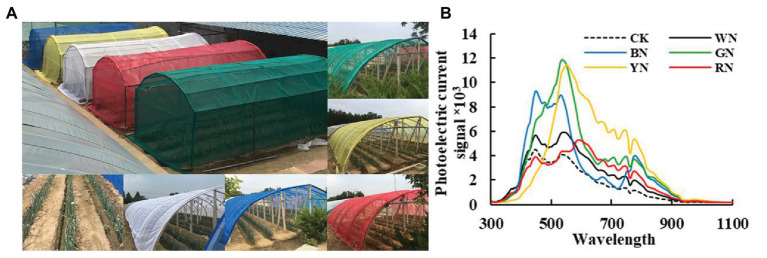
**(A)** Green onion cultivation under different photo-selective net conditions. **(B)** Spectral characteristics of each treatment. CK, control; BN, blue photo-selective nets; YN, yellow; WN, white; GN, green; and RN, red.

The experiment involved a total of six treatments. Natural light was used as a control (CK), and the five treatments included the five colors of the photo-selective nets: white (WN), blue (BN), green (GN), yellow (YN), and red (RN; shading level 30 ± 3%). The nets were arranged randomly, and each experiment was repeated three times. The photosynthetic active radiation (PAR) of each photo-selective net was recorded with HOBO Data Loggers (MicroDAQ.com, Ltd., NH, United States) to calculate the shading level = PAR_NET_/PAR_CK_ (Table 1; Figure 1A). The photo-selective nets were covered when the plants were being planted, and each treatment was managed in accordance with conventional production technology measures. Green onion plants were harvested on July 25, August 25, September 25, and October 25.

**Table 1:** Actual and target shade readings (%) for control (CK), white, blue, green, yellow, and red photo-selective netting.

Shade level	CK	White	Blue	Green	Yellow	Red
Target shade (%)	0	30	30	30	30	30
Actual shade (%)	0	28	32	29	31	30

Photosynthetic active radiation (PAR) under each replica was recorded every 30 min, from 10:00 until 18:00, and the values were compared with those in the CK to obtain the actual shading percentage. Measurements were taken at the top of the canopy.

### Spectral Irradiance Transmitted Through Photo-Selective Nets

In clear weather, the spectral characteristics of different photo-selective nets were measured using a UniSpec-SC spectrum analyzer (PP-SYSTEMS, United Kingdom), with a bandwidth of 300–1,100 nm and a 3.3 nm scanning interval. The spectral characteristics of each treatment are shown in Figure 1B.

### Analytical Methods

#### Growth Parameter, Yield, and Quality Evaluation

Plant samples were taken at the seedling stage, pseudo-stem formation stage, pseudo-stem expansion stage, and harvest stage. Three plants were selected from each treatment, and the plant height, stem diameter, leaf, and pseudo-stem fresh weight (FW), and dry weight (DW) were determined. At harvest time, plants were weighted to determine yield per plant. Supplementary Text S1 describes in detail the determination method of soluble sugar, cellulose, soluble protein, free amino acid, and vitamin C (Vc) contents.

#### Photosynthetic Pigments and Gas Exchange System

Chlorophyll (Chl) and carotenoids (CAR) were extracted and quantified using the acetone extraction colorimetric method described in Gilmore and Yamamoto (1991). First, 0.2 g of the leaf was weighted into the test tube. Twenty milliliters of 80% acetone were added and extracted in the dark for 24 h until the leaves turned white. Then, UV spectrophotometers with wavelengths set at 663 nm, 646 nm, and 470 nm were used for colorimetry. Based on the method described in Gao et al. (2020), we used the Li-6,800 portable photosynthetic apparatus (Li-COR, United States) to measure the net photosynthetic rate (Pn), stomatal conductance (Gs), intercellular CO_2_ concentration (Ci), and transpiration rate (Tr) of the third functional leaf on July 25, August 25, September 25, and October 25, and the diurnal variation in photosynthetic parameters during the pseudo-stem formation stage. Throughout the experiment, leaves of the same ontogeny were evaluated for three plants, every 2 h from 7:00 to 17:00 h (Tucci et al., 2010).

#### Chlorophyll Fluorescence

Fluorescence parameters such as initial fluorescence (Fo), maximum fluorescence (Fm), initial fluorescence (Fo'), and maximum fluorescence (Fm') were measured using an FMS-2 portable fluorometer (Hansatech, United Kingdom). The maximum photochemical efficiency (Fv/Fm), actual photochemical efficiency (ΦPSII) = (Fm'-Fs)/Fm', photochemical quenching coefficient (qP) = (Fm'-Ft)/(Fm'-Fo'), and non-photochemical quenching coefficient (NPQ) = 1-(Fm'-Fo')/(Fm-Fo) were calculated as well, the light intensity used to calculate the yield of NPQ and ΦPSII was 1,200 μmol·m^−2^·s^−1^ (Genty et al., 1989; Roháček, 2002).

#### Antioxidant System

The third functional leaves of green onion plants were selected for the assessment of the antioxidant system, including antioxidant substances and antioxidant-related enzyme activities. Malondialdehyde (MDA) content is an index used for evaluating oxidative damage and is determined by the thiobarbituric acid method (Wada et al., 2011). We used nitro blue tetrazolium (NBT) to detect the accumulation of superoxide (O_2_^−^; Xia et al., 2009). The H_2_O_2_ content was determined according to Patterson et al. (1984). The assay was based on the absorbance change of the titanium peroxide complex at 415 nm. The enzyme solution was extracted according to the method described in Gong et al. (2014). The antioxidant system was evaluated by measuring the activity of superoxide dismutase (SOD), peroxidase (POD), and catalase (CAT; Roldán et al., 2008; Chapman et al., 2019).

#### Element Contents

During the harvest period, the roots, stems, and leaves of green onion plants were washed three times with deionized water, dried at 105°C for 15 min, and then dried to a constant weight at 70°C. From each sample, 0.2 g were ground and digested with H_2_SO_4_-H_2_O_2_, and a discrete auto-analyzer (Smartchem200, Alliance, France) was used to determine N and P contents. An inductively coupled plasma optical emission spectrometer (ICP-OES; iCAP-7,400, Thermo Scientific, United States) was used to determine the potassium content in the solution.

### Data Analysis

In the present study, all plants were randomly sampled. The data were processed, plotted, and statistically analyzed in Excel 2016 and DPS software package (DPS for Windows, 2009). The differences among treatments were tested using Duncan’s new multiple range test at a significance level of *p* ≤ 0.05.

## Results

### The Shading Level and Transmission Spectrum Characteristics of Different Photo-Selective Nets

Figure 1A shows the cultivation of green onion under different photo-selective net conditions. We used PAR measurements to calculate the shadow transmittance (vs. non-netted controls). We found that compared with CK, the actual shading level of WN, BN, GN, YN, and RN were 28, 32, 29, 31, and 30%, respectively (Table 1). As shown in Figure 1B, the transmission spectra of each colored photo-selective net were different. The proportion of blue and green light in BN was significantly higher than in other treatments, the proportion of green and yellow light in GN was significantly higher than other treatments, especially green light, and the proportion of green, yellow, and red light in YN was significantly increased, and the proportion of red light in RN increased.

### Effects of Photo-Selective Nets on the Growth of Green Onion

Different light qualities and light intensities had different effects on the growth of plants at different growth stages. Table 2 shows the influence of different treatments on the growth of plants in different growth stages. There was no significant difference in plant growth among the treatments at the seedling stage. However, at the pseudo-stem formation stage, with the increase in plant height and stem diameter, the differences among the different treatments became significant. The plant height in BN and WN treatments increased by 9.74 and 3.38%, respectively, whereas in RN, GN, and YN treatments, it decreased by 2.34, 4.25, and 7.21%, respectively, compared with that in CK. The stem diameter in BN and WN treatments increased by 8.56 and 4.10%, respectively, whereas in RN, GN, and YN, it decreased by 5.96, 10.10, and 14.31%, respectively, compared with that in CK. The leaf fresh weight in BN and WN treatments increased by 6.71 and 1.57%, respectively, whereas in RN, GN, and YN, it decreased by 4.18, 5.41, and 10.87%, respectively, compared with that in CK.

**Table 2:** Effect of photo-selective nets on the growth of green onion.

Growth stage	Treatment	Plant height (cm)	Stem diameter (mm)	Leaf FW (g)	Pseudo-stem FW (g)	Aboveground FW (g)
Seedling	CK	55.50 ± 0.17ab	8.35 ± 0.01a	27.56 ± 0.11b	26.96 ± 0.08b	54.52 ± 0.18b
stage	WN	56.20 ± 0.66a	8.34 ± 0.05a	27.48 ± 0.10b	27.31 ± 0.48ab	54.79 ± 0.52b
(07–25)	BN	56.50 ± 0.30a	8.35 ± 0.02a	28.64 ± 0.98a	27.99 ± 0.16a	56.64 ± 1.10a
	GN	54.57 ± 0.90bc	8.26 ± 0.03b	25.95 ± 0.55c	25.66 ± 0.66c	51.61 ± 0.92c
	YN	54.07 ± 0.75c	8.27 ± 0.03b	25.10 ± 0.40c	25.48 ± 0.61c	50.58 ± 0.84c
	RN	53.83 ± 0.35c	8.31 ± 0.04ab	25.90 ± 0.50c	26.02 ± 0.20c	51.92 ± 0.70c
Pseudo-stem	CK	78.67 ± 0.58c	14.26 ± 0.33c	73.03 ± 0.73b	67.70 ± 1.51c	140.73 ± 1.09c
formation	WN	81.33 ± 1.53b	14.83 ± 0.10b	74.18 ± 1.19b	73.50 ± 1.59b	147.68 ± 2.30b
stage	BN	86.33 ± 1.53a	15.48 ± 0.25a	78.28 ± 0.69a	79.42 ± 2.36a	157.70 ± 3.04a
(08–25)	GN	75.33 ± 0.58d	12.82 ± 0.29e	69.08 ± 1.00c	61.84 ± 0.32e	130.92 ± 0.72e
	YN	73.00 ± 1.00e	12.22 ± 0.39f	65.09 ± 0.67d	58.43 ± 0.83f	123.52 ± 1.46f
	RN	76.83 ± 0.76 cd	13.41 ± 0.26d	69.98 ± 1.25c	64.74 ± 1.68d	134.72 ± 2.77d
Pseudo-stem	CK	100.00 ± 1.00c	19.47 ± 0.34c	104.63 ± 0.65c	101.43 ± 1.29c	206.07 ± 1.31c
expansion	WN	103.33 ± 1.53b	20.62 ± 0.56b	108.53 ± 1.34b	108.97 ± 1.97b	217.50 ± 2.59b
stage	BN	108.33 ± 1.53a	22.30 ± 0.93a	115.57 ± 1.32a	116.70 ± 2.52a	232.27 ± 1.21a
(09–25)	GN	96.33 ± 1.53d	18.37 ± 0.09d	95.30 ± 2.12e	97.67 ± 0.78d	192.97 ± 2.20e
	YN	93.00 ± 1.00e	17.88 ± 0.20d	90.17 ± 0.90f	88.83 ± 0.51e	179.00 ± 0.44f
	RN	99.00 ± 1.00c	18.76 ± 0.13 cd	102.07 ± 0.64d	97.87 ± 1.05d	199.93 ± 1.16d
Harvest	CK	111.00 ± 1.00c	25.73 ± 0.75c	133.97 ± 2.71c	142.27 ± 1.60c	276.23 ± 1.97c
stage	WN	118.33 ± 1.53b	27.00 ± 0.51b	138.10 ± 0.62b	147.57 ± 2.08b	285.67 ± 1.56b
(10–25)	BN	124.00 ± 2.00a	28.95 ± 0.84a	155.93 ± 3.19a	159.47 ± 0.61a	315.40 ± 3.30a
	GN	105.67 ± 1.53d	23.82 ± 0.36de	115.03 ± 1.66e	127.17 ± 2.02e	242.20 ± 1.95e
	YN	101.50 ± 0.50e	23.05 ± 0.61e	109.43 ± 0.74f	117.93 ± 1.86f	227.37 ± 1.45f
	RN	107.67 ± 1.53d	24.43 ± 0.33d	122.83 ± 1.78d	139.00 ± 0.85d	261.83 ± 2.54d

Values are average ± SD, *n* = 18. Different letters (a, b, c, d, e, and f) in the same column indicate significant differences among treatments at *p* ≤ 0.05 according to Duncan’s new multiple range test. FW, fresh weight; CK, control; WN, white photo-selective nets; BN, blue; GN, green; YN, yellow; and RN, red.

**Figure 2:**
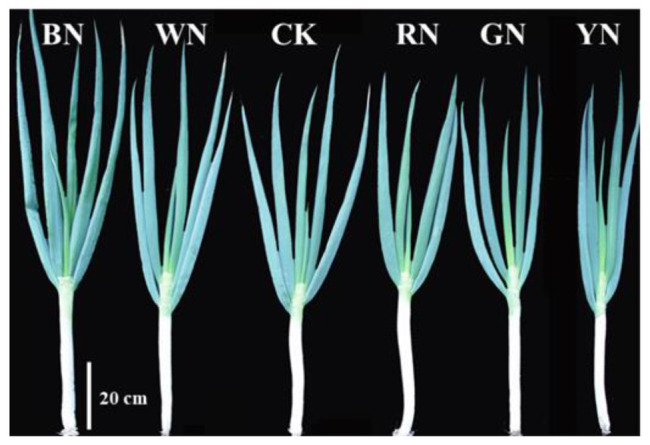
The morphology of green onion plants under different photo-selective net treatments at the harvest stage. Scale bar = 20 cm. BN, blue photo-selective nets; WN, white; CK, control; RN, red; GN, green; and YN, yellow.

At the pseudo-stem expansion stage, the pseudo-stem fresh weight varied significantly among the treatments. In BN and WN, it increased by 15.05 and 7.43%, respectively, compared with that in CK. Therefore, BN and WN treatments promoted the growth of green onion. At the harvest stage, the final aboveground fresh weight of plants in BN and WN treatments increased by 14.18 and 3.42%, respectively, whereas that in RN, GN, and YN treatments decreased by 5.21, 12.32, and 17.69%, respectively, compared with that in CK (Table 2). By measuring the height of the green onion plant, stem diameter, and the fresh weight of the plant, we found that from the best to the worst, the growth status of plants in the five treatments was BN > WN > RN > GN > YN (Figure 2).

### Effects of Photo-Selective Nets on the Yield and Quality of Green Onion

As shown in Table 3, we calculated the yield per 1000 m^2^ under different treatments, and the results showed that there was a significant difference in the yield of green onion among different treatments. Compared with the yield of CK plants, the yield of plants in BN and WN treatments increased by 14.54 and 3.77%, respectively, whereas the yields of plants in RN, GN, and YN treatments decreased by 5.47, 12.39, and 16.47%, respectively. According to quality evaluation, there was no significant difference in soluble sugar and crude cellulose content among the plants in different treatments. The contents of dry matter, soluble proteins, free amino acids, and Vc from highest to lowest were BN > WN > CK > RN > GN > YN. The dry matter, soluble protein, free amino acids, and Vc contents in plants in the BN treatment increased by 9.20, 24.83, 23.91, and 30.08%, respectively, compared with those in CK plants (Table 4).

**Table 3:** Effect of photo-selective nets on the yield of green onion.

Treatment	Plot yield (kg/200 m^2^)	Converted yield (kg/1000 m^2^)	Increase rate (%)
CK	1385.60 ± 23.08c	6928.00±115.38c	0
WN	1437.87 ± 35.25b	7189.33±176.24b	3.77
BN	1587.20 ± 27.34a	7936.00±136.70a	14.54
GN	1213.87 ± 16.42e	6069.33±82.11e	−12.39
YN	1157.33 ± 24.44f	5786.67±122.20f	−16.47
RN	1309.87 ± 35.25d	6549.33±176.24d	−5.47

Values are average ± SD, *n* = 18. Different letters (a, b, c, d, e, and f) in the same column indicate significant differences among treatments at *p* ≤ 0.05 according to Duncan’s new multiple range test. Increase rate indicates the increase in yield compared to the yield of control plants. CK, control; WN, white photo-selective nets; BN, blue; GN, green; YN, yellow; and RN, red. We measured the yield of 5 m long lengths of planted green onions in each plot, and the measurements were repeated three times for each treatment; then, we calculated the yield of each plot, and finally converted yield per 1000 m^2^.

**Table 4:** Effect of photo-selective nets on the quality of green onion.

Treatment	Dry matter (%)	Soluble proteins (mg/g)	Free amino acids (mg/g)	Vc (mg/100 g FW)	Soluble sugars (%)	Crude cellulose (mg/g)
CK	8.15 ± 0.06c	1.49 ± 0.01c	0.46 ± 0.01c	1.23 ± 0.04c	0.57 ± 0.02c	0.19 ± 0.01a
WN	8.41 ± 0.19b	1.55 ± 0.02b	0.52 ± 0.00b	1.40 ± 0.02b	0.65 ± 0.02b	0.17 ± 0.01b
BN	8.90 ± 0.05a	1.86 ± 0.02a	0.57 ± 0.00a	1.60 ± 0.02a	0.74 ± 0.01a	0.17 ± 0.01b
GN	7.73 ± 0.01d	1.45 ± 0.01c	0.45 ± 0.00d	1.02 ± 0.04e	0.55 ± 0.01c	0.15 ± 0.01c
YN	7.28 ± 0.15e	1.39 ± 0.04d	0.44 ± 0.00e	0.88 ± 0.04f	0.55 ± 0.03c	0.16 ± 0.01bc
RN	7.96 ± 0.07c	1.48 ± 0.02c	0.46 ± 0.01 cd	1.13 ± 0.04d	0.57 ± 0.04c	0.17 ± 0.01b

Values are average ± SD, *n* = 18. Different letters (a, b, c, d, e, and f) in the same column indicate significant differences among treatments at *p* ≤ 0.05 according to Duncan’s new multiple range test. CK, control; WN, white photo-selective nets; BN, blue; GN, green; YN, yellow; and RN, red.

### Effects of Photo-Selective Nets on Photosynthetic Pigments and Gas Exchange System of Green Onion

**Figure 3:**
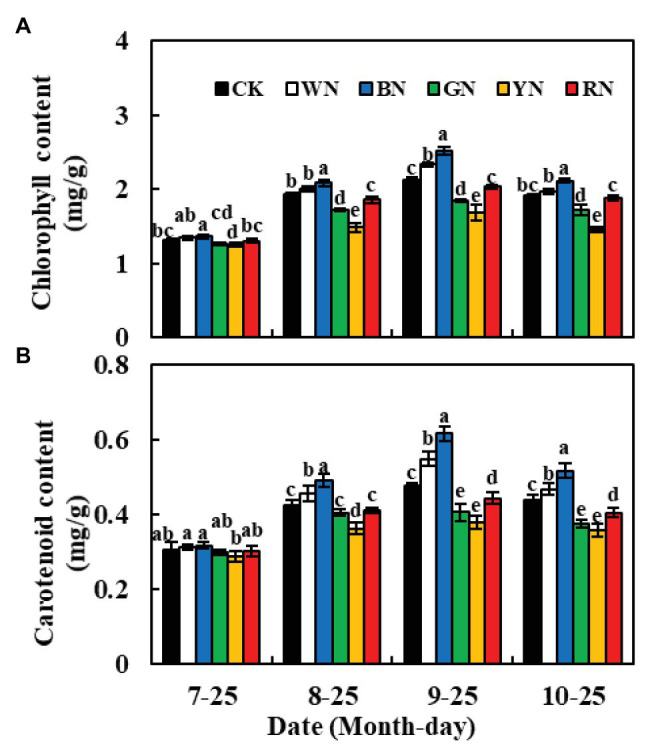
Effect of photo-selective nets on the photosynthetic pigment contents of green onion leaves at different growth stages. **(A)** Chlorophyll content, **(B)** carotenoid content. CK, control; WN, white photo-selective nets; BN, blue; GN, green; YN, yellow; and RN, red. Values are average ± SD, *n* = 18. Error bars indicate standard errors. Different letters (a, b, c, d, and e) in the same column indicate significant differences among treatments at *p* ≤ 0.05 according to Duncan’s new multiple range test.

The chlorophyll and carotenoid contents of plants in BN and WN treatments were significantly higher than those of CK plants at different growth stages, whereas, in plants in RN, GN, and YN treatments, these contents were significantly lower than those in CK plants (Figure 3). During the experiment, the chlorophyll and carotenoid contents in green onion leaves increased at first and then decreased. The highest chlorophyll content was recorded on September 25th; compared to the chlorophyll content in CK plants, the plants in BN and WN treatments had 18.87 and 10.38% higher chlorophyll content, whereas the plants in RN, GN, and YN treatments had 4.25, 12.74, and 20.75% lower chlorophyll content, respectively (Figure 3A). Therefore, both BN and WN treatments increased the pigment content in green onion leaves. The trend of change in carotenoid content was consistent with the trend of change in chlorophyll content (Figure 3B).

**Figure 4:**
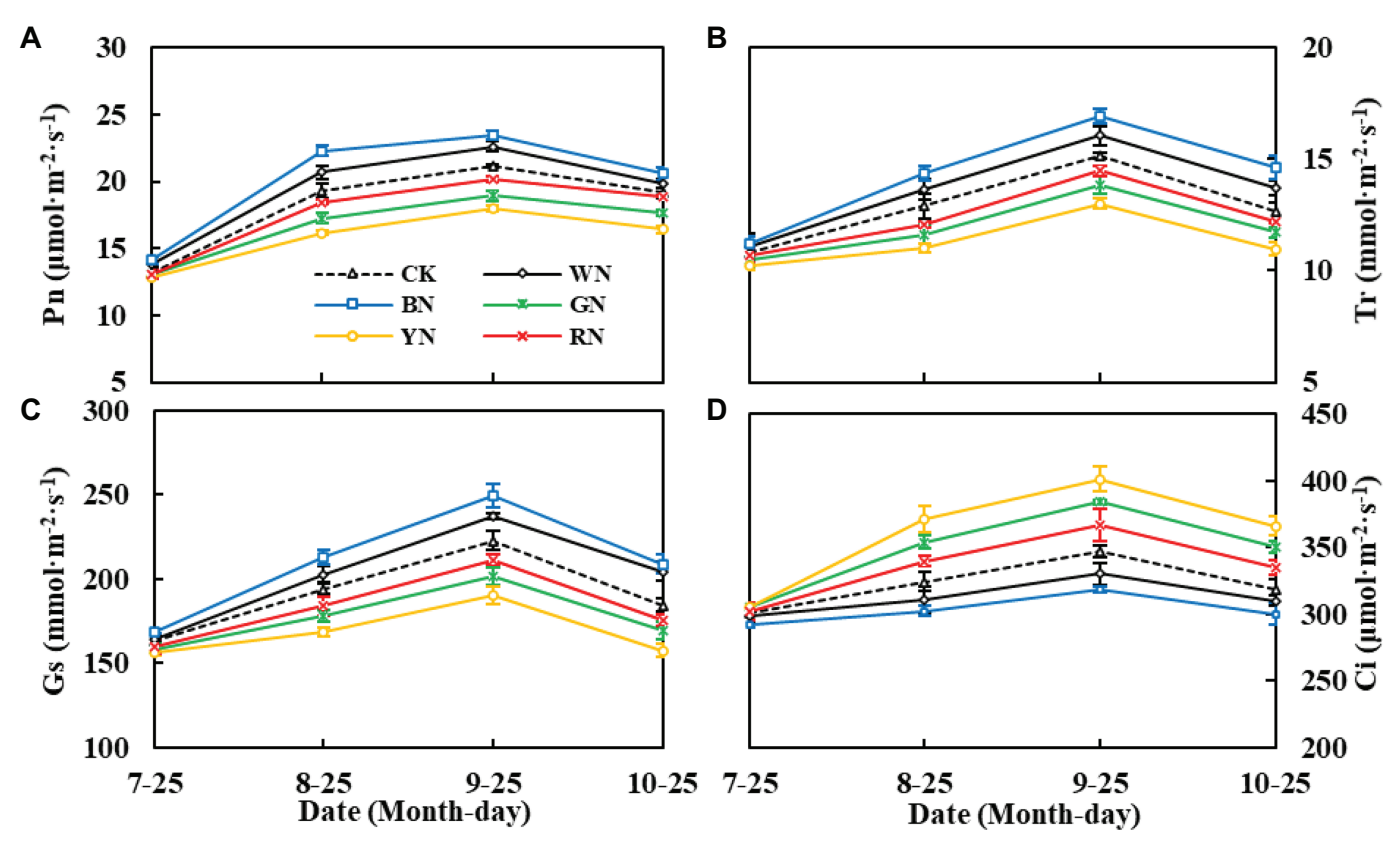
Effect of photo-selective nets on the photosynthetic parameters of green onion leaves at different growth stages. **(A)** Net photosynthetic rate (Pn), **(B)** transpiration rate (Tr), **(C)** stomatal conductance (Gs), **(D)** intercellular CO_2_ concentration (Ci). CK, control; WN, white photo-selective nets; BN, blue; GN, green; YN, yellow; and RN, red. Values are average ± SD, *n* = 18. Error bars indicate standard errors.

Figure 4 shows the effect of photo-selective nets on the gas exchange parameters of green onion leaves at different growth stages. We found that both Tr and Gs showed a trend of increasing at first and then decreasing during the growth process. The differences in Tr and Gs among the treatments were significant at the pseudo-stem formation stage and reached the highest level at the pseudo-stem expansion stage, Tr and Gs were the highest in the BN treatment (Figures 4B,C). In the pseudo-stem expansion stage on September 25th, the Pn of plants in BN and WN treatments increased by 10.86 and 6.90%, respectively, compared with that in CK, whereas in plants in RN, GN, and YN treatments, it decreased by 4.91, 10.58, and 15.12%, respectively, compared with that in CK (Figure 4A). Ci had the opposite trend than Pn, Tr, and Gs. The Ci was the highest in YN and the lowest in BN, which indicated that BN consumed more CO_2_, thus promoting Pn (Figure 4D).

**Figure 5:**
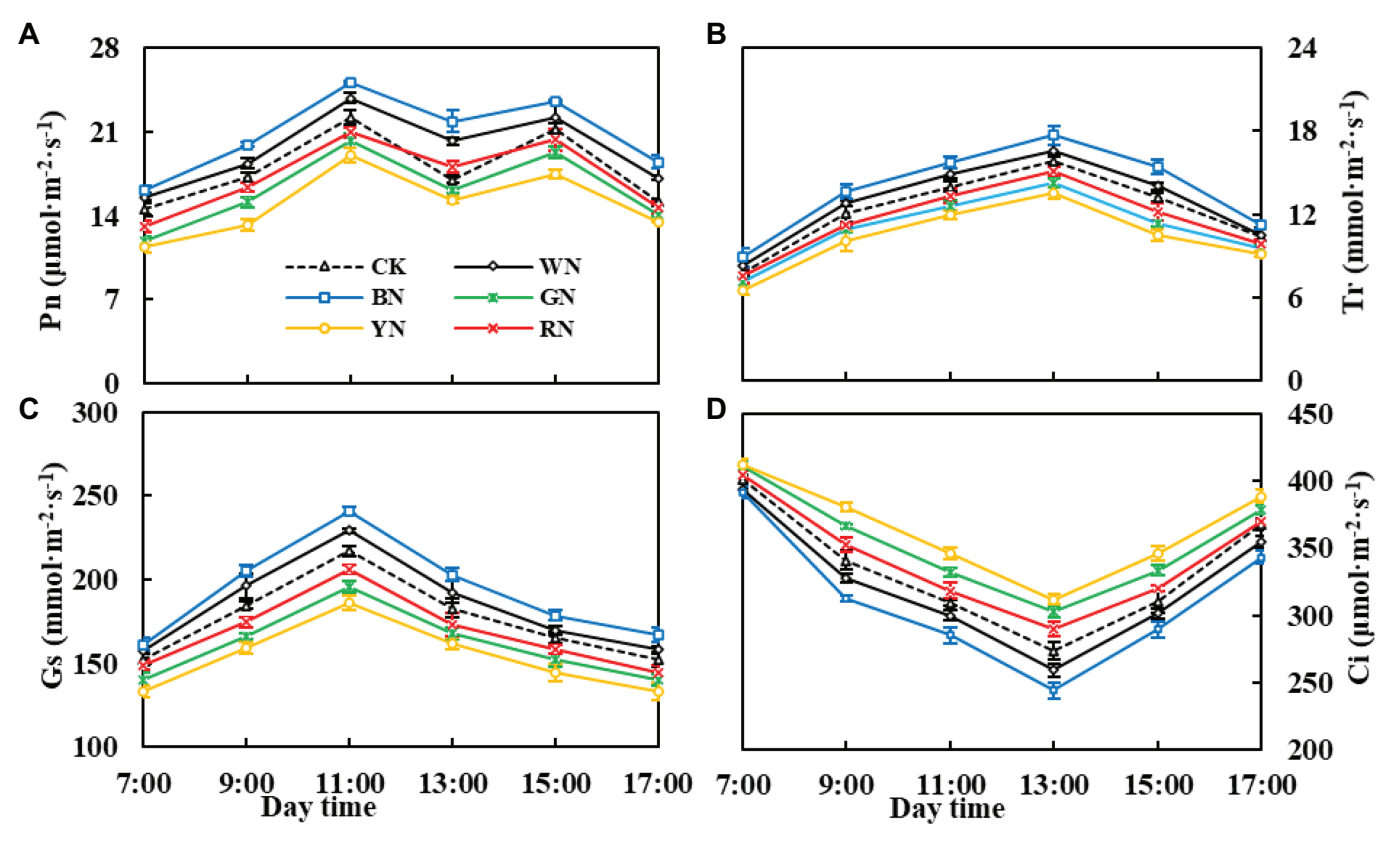
The effect of photo-selective nets on diurnal variation in photosynthetic parameters of green onion leaves during the pseudo-stem formation stage. **(A)** Net photosynthetic rate (Pn), **(B)** transpiration rate (Tr), **(C)** stomatal conductance (Gs), **(D)** intercellular CO_2_ concentration (Ci). CK, control; WN, white photo-selective nets; BN, blue; GN, green; YN, yellow; and RN, red. Values are average ± SD, *n* = 18. Error bars indicate standard errors.

By analyzing the diurnal variation in photosynthesis in the green onion during the pseudo-stem formation stage (Figures 5A–D), we found that the differences among the treatments were consistent with the trend in the growth stage, with plants in the BN treatment being the highest in Pn, Tr, and Gs. During the course of 1 day, the Pn change trend was of the “M” type: the peak values were recorded at 11:00 and 15:00, the valley was recorded at 13:00, and the “photosynthesis noon break phenomenon” (Tucci et al., 2010) in CK plants at 13:00 was more severe than that in plants under the other treatments (Figure 5A). Compared with that in CK plants, the Pn of plants in BN, WN, and RN treatments increased by 28.60, 18.79, and 6.46%, respectively. Therefore, covering with photo-selective nets could enhance the hardiness of the green onion leaves, improve the leaf net photosynthetic rate, and reduce the occurrence of “photosynthesis noon break phenomenon.” The diurnal variation in Tr and Gs of green onion leaves in different treatments showed a single peak curve. There was a significant difference in Tr and Gs between the treatments at 11:00, and Tr peaked at 13:00 (Figure 5B). The diurnal variation in Gs with the trend of Pn reached the maximum at 11:00 and then declined (Figure 5C). The diurnal variation in Ci was characterized by the highest values in the morning, followed by those in the afternoon, and the lowest values at noon, which was essentially the opposite of Pn (Figure 5D).

### Effects of Photo-Selective Nets on Chlorophyll Fluorescence of Green Onion

**Figure 6:**
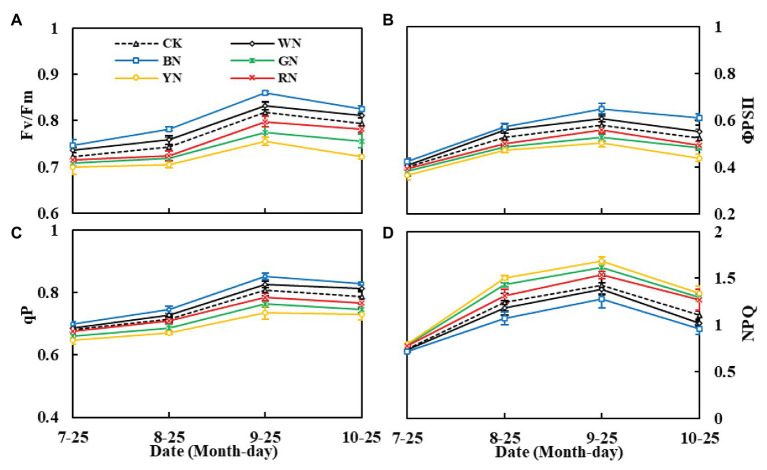
The effect of photo-selective nets on the fluorescence parameters of green onion leaves at different growth stages. **(A)** Maximum photochemical efficiency of PSII under dark adaptation (Fv/Fm), **(B)** actual photochemical efficiency (ΦPSII), **(C)** photochemical quenching coefficient (qP), **(D)** non-photochemical quenching coefficient (NPQ). CK, control; WN, white photo-selective nets; BN, blue; GN, green; YN, yellow; and RN, red. Values are average ± SD, *n* = 18. Error bars indicate standard errors.

Furthermore, photo-selective nets significantly affected the chlorophyll fluorescence parameters of green onion leaves. From highest to lowest, the Fv/Fm, qP, and ФPSII of each treatment were BN > WN > CK > RN > GN > YN. Moreover, throughout the growth period, from the seedling stage to the pseudo-stem expansion stage, the parameters showed an upward trend, and the indicators from the pseudo-stem expansion stage to the harvest stage declined, whereas the trend of NPQ was the opposite (Figures 6A–D). In the pseudo-stem expansion stage, compared with that in CK plants, the Fv/Fm of plants in BN and WN treatments increased by 4.88 and 1.84%, whereas in RN, GN, and YN, it decreased by 2.33, 5.14, and 7.47%, respectively. The results showed that BN and WN improved the light energy conversion efficiency of the PSII reaction center and enhanced the actual light energy capture efficiency when this reaction center was partially closed, whereas YN and GN increased the heat dissipation of green onion plants.

### Effects of Photo-Selective Nets on the Antioxidant System of Green Onion

**Figure 7:**
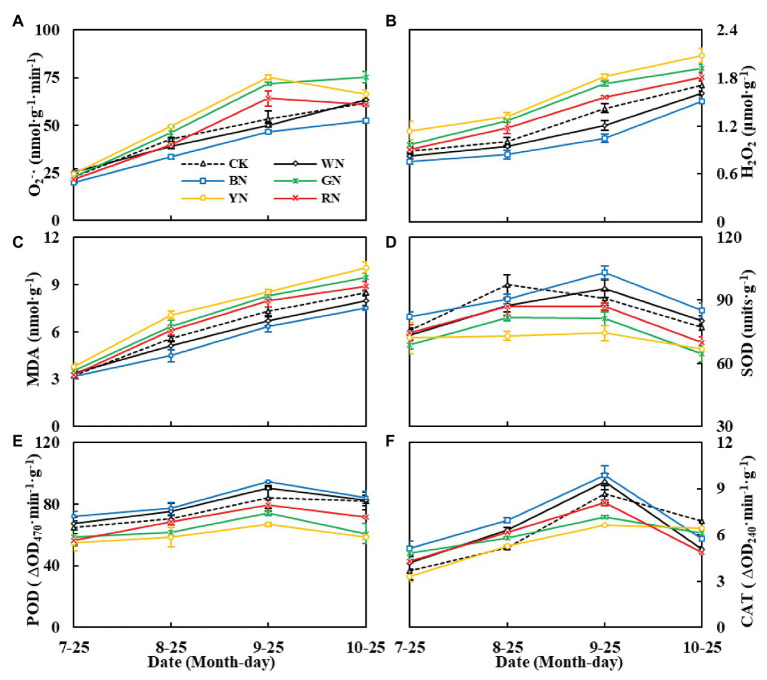
The effect of photo-selective nets on reactive oxygen, malondialdehyde (MDA) contents, and antioxidant enzyme activities of green onion leaves at different growth stages. **(A)** O_2_^−^ (superoxide anion), **(B)** H_2_O_2_, **(C)** MDA content, **(D)** SOD activity, **(E)** POD activity, **(F)** CAT activity. CK, control; WN, white photo-selective nets; BN, blue; GN, green; YN, yellow; and RN, red. Values are average ± SD, *n* = 18. Error bars indicate standard errors.

As shown in Figure 7, the level of reactive oxygen species (ROS) in the leaves of plants under photo-selective nets continued to increase with the increase in plant growth. The levels of ROS in YN, GN, and RN treatments were significantly higher than those in the CK treatment (Figures 7A,B), the MDA content was consistent with the trend of ROS levels (Figure 7C). In plants, superoxide dismutase (SOD) is an important protective enzyme for removing ROS. It can effectively remove excess O_2_^−^ and decrease its peroxidation effect on membrane lipids. In the present study, the activity of SOD in green onion leaves showed an upward trend followed by a downward trend during the experiment, with the highest activity on September 25. At that time, the SOD activity in the leaves of plants in BN and WN treatments was 13.34 and 4.85% higher than that in the leaves of CK plants, respectively, and in the leaves of plants in RN, GN, and YN treatments, it was 4.50, 10.90, and 18.46% lower than that in the leaves of CK plants, respectively. The trend of POD and CAT activity was similar to that of SOD activity (Figures 7D–F). BN and WN reduced the accumulation of ROS by increasing the activity of SOD, POD, and CAT to further decrease MDA levels in leaves.

### Effects of Photo-Selective Nets on the Nutrient Content of Green Onion

**Figure 8:**
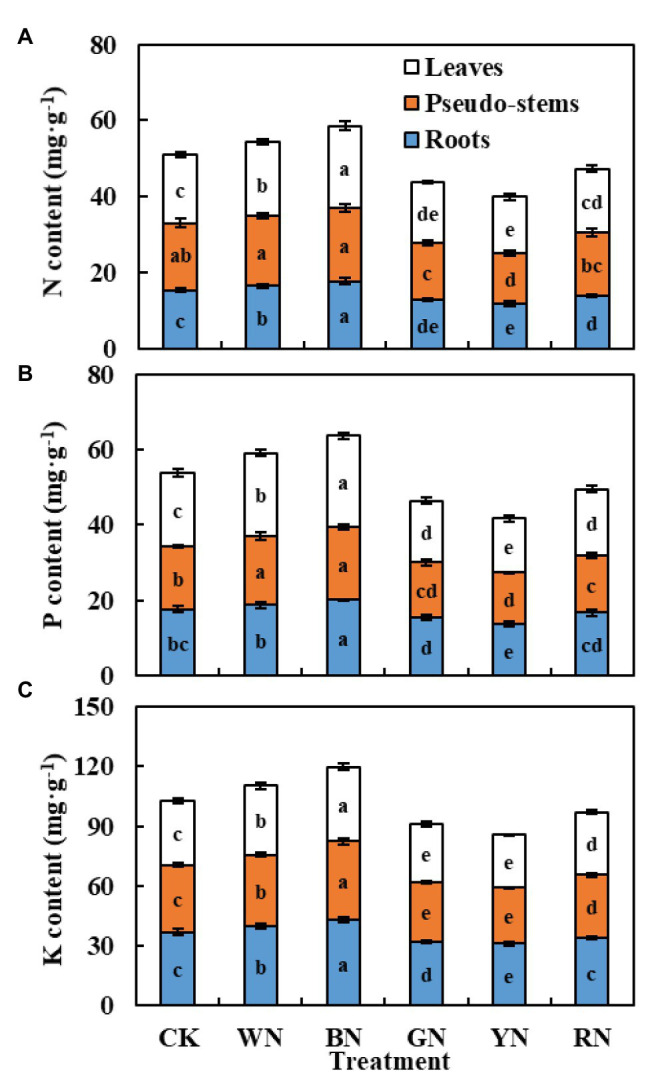
The effect of photo-selective nets on element content of green onion leaves, pseudo-stems, and roots during the harvest stage. **(A)** N content, **(B)** P content, **(C)** K content. CK, control; WN, white photo-selective nets; BN, blue; GN, green; YN, yellow; and RN, red. Values are average ± SD, *n* = 18. Error bars indicate standard errors. Different letters (a, b, c, d, and e) in the same column indicate significant differences among treatments at *p* ≤ 0.05 according to Duncan’s new multiple range test.

Macroelements refer to the essential nutrients required for normal plant growth and development. In our experiment, we mainly determined the contents of N, P, and K in the roots, pseudo-stems, and leaves of green onion during the harvest stage. We found that the balance of nutrients in plants was significantly different under different photo-selective nets. Figure 8 summarizes the contents of various mineral elements in the roots, stems, and leaves of green onion under different treatments. We found that the nitrogen content in roots, pseudo-stems, and leaves was the highest under the BN treatment, and the trend among treatments was BN > WN > CK > RN > GN > YN. The changing trend of phosphorus and potassium content was consistent with nitrogen content.

## Discussion

Photo-selective nets are considered to prevent excessive sun radiation, improve soil moisture, stabilize air temperature, and promote normal plant growth. They can also change the radiation spectrum available to plants, which results in metabolic adjustment of the photosynthesis system (Sabino et al., 2016), which may help improve the plant growth characteristics evaluated in the present study. In a study carried out in Hungary using colored shading nets on peppers, the temperature changed relatively little in response to the nets, and it decreased as the shading level increased (Ombódi et al., 2015). It was reported that coffee trees planted under red nets have higher biomass than those planted under other types of shade nets (Henrique et al., 2011). This may be because red shading nets allowed the plants to absorb more photons of red wavelength, which may be beneficial for increasing the activity of photosynthetic pigments, resulting in increased photosynthetic assimilation (Yu et al., 2017). In our experiment, plant height, stem diameter, and aboveground fresh weight of plants under BN and WN treatments were significantly higher than those in plants under CK, RN, GN, and YN treatments (Table 2). This was inconsistent with the results of previous research, suggesting possible species-specific variation. It is possible to modify desirable plant growth characteristics by combining light scattering and spectral manipulation.

Light conditions have an important impact on the quality and yield of vegetable products (Fu et al., 2017). Red and pearl nets have been shown to improve the quality of tomato fruits (Ilić et al., 2012, 2015). Compared with traditional black nets, light adjustment and microclimate conditions under pearl nets provided higher crop yields (Ilić et al., 2011). Pearl nets not only improved the yield and quality of sweet peppers but also improved the postharvest shelf life of sweet pepper fruits (Selahle et al., 2015; Díaz-Pérez et al., 2020). In our experiment, BN increased the content of dry matter, soluble proteins, free amino acids, Vc, and soluble sugars (Table 4). This may be because BN improved the yield (Table 3) by improving the water use efficiency and absorption and conversion of various elements (Figure 8).

Chlorophyll is the basis for photosynthesis in plants. The chlorophyll content of plants in BN and WN treatments was significantly higher than that of CK plants. Ilić et al. (2017a) also found that the total chlorophyll content of leaves of lettuce cultivated under blue and black nets was higher than that of leaves of lettuce cultivated under other nets. Carotenoids act as antenna pigments and transmit the absorbed light energy to chlorophyll for further photochemical reaction, and they also serve to protect the chlorophylls from too much light or the wrong light wavelengths and thus act as a selective filter (Ilić et al., 2017b). This may be the reason why Pn was higher in plants under the BN treatment than in plants under the other treatments. Blue light has been proven to promote stomata opening (Inoue et al., 2008). In our experiment, the Gs of plants in the BN treatment were significantly higher than those of plants in the other treatments, which may also be the reason for the higher Tr. Plants with higher Pn consumed more CO_2_, which led to a decrease in the leaf Ci concentration (Figure 4). The increase in Tr compared to the Tr of CK plants indicated that BN could improve the absorption efficiency of nutrients by plants and allocate more nutrients to the aboveground organs. Increased Gs can lead to an increase in Ci concentration, which is beneficial for improving the photosynthetic activity of leaves and promoting the increase in Pn. From the diurnal variation in photosynthetic parameters (Figure 5), it can be seen that covering the plants with BN reduced the noon photosynthetic inhibition. This may be because BN can improve plant performance by reducing the noon photosynthetic inhibition caused by high temperatures and stomatal closure (Raveh et al., 2003).

In our study, taking the pseudo-stem expansion stage as an example, the Fv/Fm, ΦPSII, and qP of plants in the BN and WN treatments were significantly higher than those of CK plants, whereas Fv/Fm was significantly reduced and NPQ increased in plants in the GN and YN treatments compared to that in plants in the other treatments. This indicated that GN and YN caused a severe decrease in the photosynthesis of green onion plants and an increase in the heat dissipation in LHCII. In addition, this mainly indicated that green onion grown under GN and YN responded to photoinhibition; this can cause the production of ROS, which also explained the higher ROS content in green onion leaves in the YN treatment than in green onion leaves in the other treatments.

Oxidative stress can lead to the inhibition of photosynthesis and respiration processes, thereby inhibiting plant growth. MDA is one of the peroxidation products of cell membranes, reflecting the antioxidant capacity of plants. As important protective enzymes of the active oxygen scavenging enzyme system in plants, the increase in SOD, CAT, and POD activity could effectively prevent the accumulation of active oxygen, thereby ensuring that the MDA content was maintained at a low level, and maintaining normal plant growth. SOD is the first line of defense against oxidative stress and lipid peroxidation, catalyzing O_2_^−^ to generate H_2_O_2_ and O_2_, whereas POD and CAT can remove H_2_O_2_ from peroxisomes and the cytoplasm (Dewir et al., 2006; Wu, 2016). In the present study, the contents of O_2_^−^, H_2_O_2_, and MDA in plants under BN and WN were lower, whereas the activities of SOD, POD, and CAT were higher than those of plants under the other photo-selective nets (Figure 7), indicating that BN and WN could effectively improve antioxidant capacity and delay the senescence of green onion plants.

## Conclusion

This study found that photo-selective nets, especially blue and white nets, can affect the growth of green onion by increasing the antioxidant enzyme activity, chlorophyll content, photosynthetic performance, and element absorption and transformation capabilities of green onion. In green onion cultivation, blue nets can be used to improve plant yield and quality.

## Data Availability Statement

The original contributions presented in the study are included in the article/Supplementary Material, further inquiries can be directed to the corresponding author.

## Author Contributions

SG gathered samples, participated in the study design, performed data analysis, interpreted the results, and drafted the manuscript. XL and YL conducted experiments. KX conceived of the study, provided funding, and gave guidance on experimental design. KX, BC, and ZC modified the paper. All authors contributed to the article and approved the submitted version.

### Conflict of Interest

The authors declare that the research was conducted in the absence of any commercial or financial relationships that could be construed as a potential conflict of interest.
