# Supplementary material for: The Spectral Irradiance, Growth, Photosynthetic Characteristics, Antioxidant System, and Nutritional Status of Green Onion (Allium fistulosum L.) Grown Under Different Photo-Selective Nets
Source: Front Plant Sci. 2021 Mar 25;12:650471. doi: 10.3389/fpls.2021.650471 (PMC8030602; doi:10.3389/fpls.2021.650471)
Supplement: Supplementary file 1 [file Presentation_1.pdf]

The spectral irradiance, growth, photosynthetic characteristics, antioxidant system, and nutritional status of green onion (*Allium fistulosum* L.) grown under different photo-selective nets

Song Gao[a, b, c, d], Xuena Liu[a, b, c, d], Ying Liu[a, b, c, d], Bili Cao[a, b, c, d], Zijing Chen[a, b, c, d] *, Kun Xu[a, b, c, d,] *

[a] *College of Horticulture Science and Engineering, Shandong Agricultural University*

[b] *Collaborative Innovation Center of Fruit & Vegetable Quality and Efficient Production in Shandong*

[c] *Key Laboratory of Biology and Genetic Improvement of Horticultural Crops in Huanghuai Region, Ministry of Agriculture and Rural Affairs, P. R. China*

[d] *State Key Laboratory of Crop Biology, Tai'an 271018, China*

[*] Corresponding author E-mail addresses: xukun@sdau.edu.cn (K. Xu); chenzijing@sdau.edu.cn (Z. Chen)

Text S1

Soluble sugar. A 0.5 g sample of green onion leaf and pseudo-stem homogenate was accurately weighed, 10 ml of distilled water was added to the homogenate, and centrifuge at 10,000 × g for 10 min using a centrifuge. Then, the soluble sugar content was determined as described by Wan et al. (2014).

Cellulose. The cellulose content was determined by concentrated sulfuric acid hydrolysis and sugar determination (Viles and Silverman, 1949) with slight modifications. Samples of 0.5 g of green onion leaf and pseudo-stem were accurately weighed and then digested with 60 ml of 60% sulfuric acid solution in a cold-water bath for 30 min. The mixture was brought to 100 ml with 60% sulfuric acid solution, shaken well, and filtered with a Buchner funnel. Then, 5 ml of the filtrate was placed in a 100 ml volumetric flask, shaken, brought to 100 ml with distilled water, and filtered. The filtrate was used as the cellulose extract to determine the cellulose content.

Soluble protein. The soluble protein content was measured according to Paciolla et al. (2010) with slight modifications. One gram of frozen green onion was ground in 4 ml of a solution containing the following ingredients: 0.05 M Tris-HCl (pH 8.0), 0.001 M EDTA, 0.01 M $MgCl_2$ and 0.05% cysteine. The homogenate was centrifuged at 20,000 × g for 20 min at 4 °C, and the resulting supernatant was tested for soluble protein content according to Bradford (1976) with serum albumin as the standard.

Free amino acid. The free amino acid content was measured by the ninhydrin solution chromogenic method (Huang et al., 2010). Green onion leaves and pseudo-stem powder (0.4 g) were weighed out separately, placed in a 250 mL round-bottom flask, and boiled with water 3 times. Added 40 ml of water, boil for 1 h, added 20 mL of water, boil for 0.5 h, added 20 mL of water, boil for 0.5 h, and filtered while hot after each boiling. The three filtrates were combined and placed in a 100 mL volumetric flask, diluted with water to 100 mL, and used as the test solution. Three milliliters of the test solution was placed in a 25 mL volumetric flask, 1.0 mL of sodium acetate buffered saline solution at pH 5.0 and 2.0 mL of 2.0% ninhydrin solution were added, and the solution was boiled in a 100 °C

water bath for 19 min and then cooled for 10 min. The absorbance of the color system was measured at 568 nm. The free amino acid content was calculated according to the standard curve of Huang et al (2010).

Vitamin C. Vitamin C content was assessed spectrophotometrically (Samuoliene et al., 2012). Plant tissue (1.0 g) was accurately weighed out, and 10 ml of 5% oxalic acid was added to grind it into a homogenate. Then centrifuged at 2000 × g for 5 min at 4 °C. Add 2 ml of 0.1% methyl viologen and 2 ml of 2 M sodium hydroxide to 1 ml of supernatant, shake well, and let stand for 2 min. The OD was read at 600 nm with a spectrophotometer.

Bradford, M.M., 1976. A rapid and sensitive method for the quantitation of microgram quantities of protein utilizing the principle of protein-dye binding. Anal. Biochem. 72, 248-254. doi: https://doi.org/10.1016/0003-2697(76)90527-3

Huang, S., Wu, Y., Liu, M., 2010. Quantitative determination of total free-amino acid in Nervilia fordii (Hance) Schltr. by ninhydrin colorimetric method. Chinese Journal of Information on Traditional Chinese Medicine 17, 50-52. doi: 10.3969/j.issn.1005-5304.2010.12.021

Paciolla, C., D'Emerico, S., Tommasi, F., Scrugli, A., 2010. Karyomorphological and biochemical studies in Glebionis coronaria (L.) Spach and Glebionis segetum (L.) Fourreau from Italy. Plant Biosystems - An International Journal Dealing with all Aspects of Plant Biology 144, 563-567. doi: 10.1080/11263501003658438

Samuoliene, G., Sirtautas, R., Brazaityte, A., Duchovskis, P., 2012. LED lighting and seasonality effects antioxidant properties of baby leaf lettuce. Food Chem. 134, 1494-1499. doi: https://doi.org/10.1016/j.foodchem.2012.03.061

Schwimmer, S., Weston, W., 1961. Onion flavor and odor, enzymatic development of pyruvic acid in onion as a measure of pungency. J. Agric. Food Chem. 9, 301-304. doi: 10.1021/jf60116a018

Tsakaldimi, M., Ganatsas, P., Jacobs, D.F., 2012. Prediction of planted seedling survival of five Mediterranean species based on initial seedling morphology. New Forests 44, 327-339. doi: 10.1007/s11056-012-9339-3

Viles, F.J., Silverman, L., 1949. Determination of Starch and Cellulose with Anthrone. Analytical Chemistry 21, 950-953. doi: 10.1021/ac60032a019

Wan, Y.-Y., Zhang, Y., Zhang, L., Zhou, Z.-Q., Li, X., Shi, Q., Wang, X.-J., Bai, J.-G., 2014. Caffeic acid protects cucumber against chilling stress by regulating antioxidant enzyme activity and proline and soluble sugar contents. Acta Physiologiae Plantarum 37, 1706. doi: 10.1007/s11738-014-1706-6
